# Establishment of reference intervals for plasma IL-6, IL-8, IL-10, and IL-1β in healthy adults from Lianyungang, Jiangsu, China: a single-center flow cytometry analysis

**DOI:** 10.3389/fmed.2026.1789886

**Published:** 2026-05-13

**Authors:** Fumeng Yang, Ruoxue Cao, Fang Yang, Qian Liu, Wei Zhu

**Affiliations:** 1 School of Medicine, Jiangsu University, Zhenjiang, China; 2 Department of Laboratory Medicine, Affiliated Lianyungang Clinical College of Nantong University, Lianyungang, China

**Keywords:** cytokines, flow cytometry, IL-10, IL-1β, IL-6, IL-8, reference intervals

## Abstract

**Background:**

Cytokines are central regulators of both physiological and pathological immune responses. In this study, flow cytometry was used to quantify plasma interleukin-6 (IL-6), interleukin-8 (IL-8), interleukin-10 (IL-10), and interleukin-1β (IL-1β), with the aim of establishing reference intervals to support clinical interpretation, diagnosis, and treatment.

**Methods:**

Between July and November 2025, 728 healthy adults from Jiangsu Province in eastern China were enrolled as reference individuals according to predefined inclusion and exclusion criteria. Plasma IL-6, IL-8, IL-10, and IL-1β concentrations were measured by flow cytometry. Data distribution was evaluated using the Kolmogorov-Smirnov test. In accordance with the Clinical and Laboratory Standards Institute (CLSI) C28-A3 and WS/T 402–2024, nonparametric reference intervals were established using the 95th percentile.

**Results:**

Plasma IL-6, IL-8, IL-10, and IL-1β concentrations showed non-normal distributions. No significant differences were observed by sex or age (all *P* > 0.05); therefore, the entire cohort was analyzed as a single reference population. The resulting reference intervals, with 90% confidence intervals (CIs), were IL-6 ≤ 2.23 pg/mL (90% CI: 2.18–2.27 pg/mL), IL-8 ≤ 3.76 pg/mL (90% CI: 3.51–3.98 pg/mL), IL-10 ≤ 1.89 pg/mL (90% CI: 1.81–2.02 pg/mL), and IL-1β ≤ 1.34 pg/mL (90% CI: 1.17–1.39 pg/mL).

**Conclusion:**

To our knowledge, this is the first study to establish reference intervals for plasma IL-6, IL-8, IL-10, and IL-1β in a healthy adult population from Lianyungang, Jiangsu, eastern China. These intervals may provide a useful basis for evaluating immune status and for supporting the diagnosis and management of related clinical conditions.

## 1 Introduction

Cytokines are small soluble proteins that mediate intercellular communication and are secreted by immune cells as well as tissue-resident cells (1). Among them, interleukin-6 (IL-6), interleukin-8 (IL-8), interleukin-10 (IL-10), and interleukin-1β (IL-1β) are particularly important in immune regulation and disease pathogenesis (2–5). These mediators participate in a wide range of physiological and pathological processes and have been implicated in inflammatory disorders, malignancies, coronary artery disease, infections, and autoimmune diseases (6–10). Accordingly, in vitro measurement of cytokines can help assess immune status and assist clinical decision-making.

In laboratory medicine, reference intervals are essential for interpreting test results and guiding diagnosis and treatment (11). However, formally established reference intervals for plasma cytokines remain limited, and many laboratories still rely on manufacturer-provided values that have not been independently verified (12). Because reference intervals are influenced by geography, population characteristics, and analytical methodology, they should be evaluated carefully before routine application (13). Use of inappropriate intervals may lead to misinterpretation of results, inaccurate diagnosis, and suboptimal treatment decisions (13). Therefore, laboratories should establish and validate reference intervals for commonly used cytokines, including IL-6, IL-8, IL-10, and IL-1β, based on local populations and specific assay platforms.

In the present study, plasma IL-6, IL-8, IL-10, and IL-1β concentrations were measured in healthy adults by flow cytometry. The distributions of these cytokines were examined according to age and sex. Following the Clinical and Laboratory Standards Institute (CLSI) C28-A3 and WS/T 402–2024 (14, 15), we established reference intervals for a healthy adult population from Lianyungang, Jiangsu, eastern China. The resulting intervals are intended to improve the clinical utility of cytokine testing in preventive screening, disease diagnosis, and therapeutic monitoring.

## 2 Materials and methods

### 2.1 Study subjects

**FIGURE 1:**
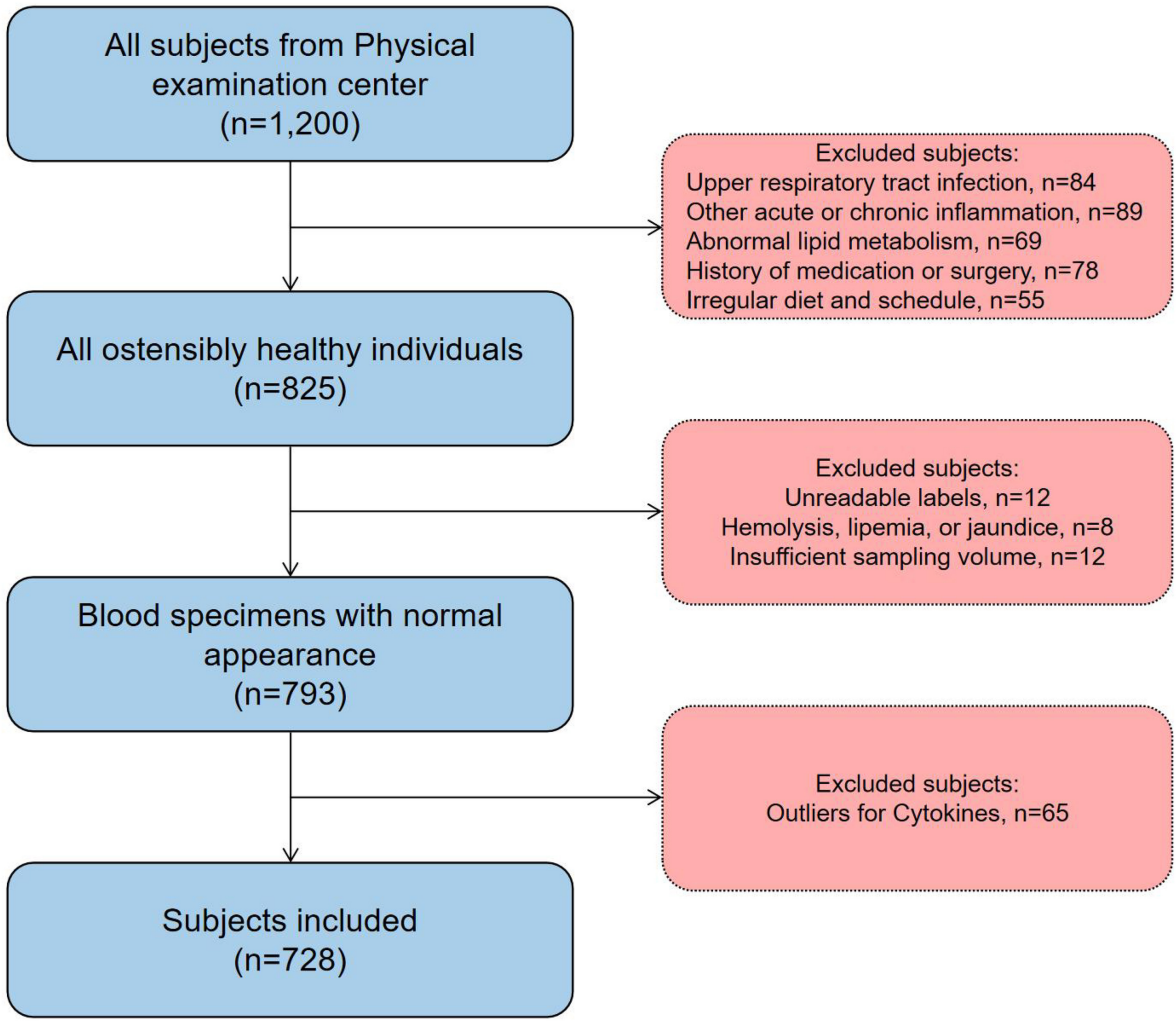
Flowchart of participant screening.

Between July and November 2025, 1,200 potential participants were recruited by simple random sampling from the Physical Examination Center of the Second People’s Hospital of Lianyungang. After the predefined inclusion and exclusion criteria were applied, 728 individuals were eligible for enrollment. The final study population comprised 364 men and 364 women aged 20–79 years. A flow diagram of participant screening is shown in Figure 1.

Eligible participants met all of the following criteria: (1) age 20–79 years and body mass index (BMI) 18.5–27.9 kg/m^2^; (2) normal blood pressure (systolic 90–140 mmHg, diastolic 60–90 mmHg); (3) hematological and biochemical indices, including white blood cell count (WBC), C-reactive protein (CRP), liver and renal function, glucose, and lipid parameters, within established reference ranges; (4) negative serological results for hepatitis B virus, hepatitis C virus, syphilis, and Human Immunodeficiency Virus (HIV); and (5) no clinically significant abnormalities on physical examination, chest radiography, electrocardiography, or abdominal ultrasonography.

Participants were excluded if they had any of the following: (1) recent surgery or medication use; (2) recent blood donation, transfusion, or major blood loss; (3) chronic exposure to physical or chemical hazards, such as ionizing radiation, benzene, or lead; (4) heavy alcohol consumption, either chronic or within the previous 2 weeks, or smoking more than 20 cigarettes per day; (5) pregnancy or lactation; (6) hematological disease, including anemia, leukemia, or thrombocythemia; (7) active allergic disorders, such as urticaria, asthma, or allergic dermatitis; (8) respiratory disease, including infection, tuberculosis, or chronic obstructive pulmonary disease (COPD); (9) urinary system disease; (10) digestive system disease; (11) autoimmune or rheumatic disease; (12) thyroid dysfunction; (13) known parasitic infection; or (14) any history of malignancy.

The study was approved by the Medical Ethics Committee of the Second People’s Hospital of Lianyungang (2025K084), and written informed consent was obtained from all participants.

### 2.2 Study design

According to CLSI C28-A3 and WS/T 402–2024 (14, 15), participants were first stratified by sex. If a significant sex-related difference was observed (*P* < 0.05), further age stratification was performed within each sex group using the following categories: 20–29, 30–39, 40–49, 50–59, and ≥ 60 years. If no significant sex-based difference was found (*P* ≥ 0.05), all participants were pooled and then categorized only by age using the same age groups. Reference intervals for plasma IL-6, IL-8, IL-10, and IL-1β were subsequently established on the basis of this stratification strategy.

### 2.3 Sample collection and processing

For 3 days before blood sampling, participants were instructed to maintain their usual diet and daily routine. After an overnight fast, 14 mL of morning venous blood was collected from each participant.

Samples were distributed into two 2-mL EDTA-K2 anticoagulant tubes and two 5-mL plain tubes. These specimens were used for cytokine measurement and for routine hematological, biochemical, and immunological testing.

When biochemical parameters were analyzed using automated instruments, the fully automated analyzer was able to identify abnormal specimens, such as hemolyzed, lipemic, or icteric samples. Such unsuitable specimens were excluded on the basis of the corresponding instrument-generated parameters.

EDTA-K2 anticoagulated specimens were centrifuged within 30 min after collection (centrifugal force: 1,000 g; duration: 10 min; temperature: 25°C). The separated plasma was analyzed within 4 h. All samples collected on the same day were processed in a single analytical batch. For each batch, third-party quality control materials (low and high levels) were used for internal quality control to ensure that assay conditions remained consistent with the test samples, thereby allowing continuous monitoring of system stability and inter-batch variation. The laboratory applied multi-rule criteria (1_3s_/2_2s_/R_4s_/4_1s_) for interpreting internal quality control results. Violation of any of these rules was considered an out-of-control event. After such an event, effective corrective actions were implemented before sample analysis proceeded, ensuring that the analytical system met the required performance specifications.

### 2.4 Instruments and reagents

Plasma IL-6, IL-8, IL-10, and IL-1β concentrations were measured using a DxFLEX flow cytometer (Beckman Coulter, Brea, CA, United States) and matching commercial kits (Qingdao Raisecare Biotechnology Co., Ltd., Qingdao, China; lot 240511), according to the manufacturer’s instructions. WBC and CRP were measured using a BC-7500CRP automated hematology analyzer (Shenzhen Mindray Biomedical Electronics Co., Ltd., Shenzhen, China). Biochemical analytes, including alanine aminotransferase (ALT; lot AUZ3365), aspartate aminotransferase (AST; lot AUZ3727), urea (lot AUZ4364), creatinine (lot 2574), glucose (GLU; lot AUZ3936), total cholesterol (TC; lot AUZ4150), and triglycerides (TG; lot AUZ4052), were measured on an AU5800 automated biochemical analyzer (Beckman Coulter, Brea, CA, United States).

### 2.5 Analytical performance characteristics of the cytokine assays

A commercial cytokine assay kit was used in this study. Our laboratory performed an in-house verification of the performance claims provided by the manufacturer in accordance with YY/T 1789.3–2022, CNAS-GL037, CNAS-GL047, and WS/T 408–2024 (16–19). The evaluated parameters included the limit of blank (LoB), limit of detection (LoD), lower limit of quantification (LLoQ), intra-assay and inter-assay precision, trueness, linearity, and reportable range. The verification results are briefly summarized as follows: LoB, 0.20 pg/mL; LoD, 0.50 pg/mL; LLoQ, 1.00 pg/mL; intra-assay precision ≤ 8.00%; inter-assay precision ≤ 12.00%; trueness ≤ 15.00%; linearity, 1.00–10,000.00 pg/mL; and reportable range, 1.00–20,000.00 pg/mL. The gating strategy for beads was performed using forward scatter and side scatter combined with fluorescence channels. For each cytokine, a minimum of 100 bead events were gated.

All verified results were consistent with the manufacturer’s claims. Detailed data are provided in Supplementary Table 1.

### 2.6 Cytokine assay principle and protocol

Cytokines were measured using a direct sandwich immunoassay. Briefly, target cytokines in samples or calibrators were captured by antibodies coupled to fluorescent microspheres. A biotinylated detection antibody then bound to the captured cytokine to form an antibody-cytokine-antibody sandwich complex. This complex was subsequently incubated with phycoerythrin-labeled streptavidin. Finally, the fluorescence intensity of each bead population was measured by flow cytometry, allowing quantification of cytokine concentrations. All procedures were performed strictly in accordance with the manufacturer’s instructions.

### 2.7 Validation of reference intervals

In accordance with WS/T 402–2024 (15), the established reference intervals were validated using a relatively large validation sample (*n* = 60). The validation cohort was independent of the reference cohort and was enrolled in December 2025.

The same inclusion and exclusion criteria were applied to both cohorts, and the procedures for specimen collection, pre-analytical handling, and measurement were kept consistent to minimize heterogeneity related to workflow differences.

(1) Sixty eligible reference individuals who met the criteria described in section 2.1 were enrolled as the validation cohort. Any outliers identified during testing were excluded and replaced as necessary so that the final sample size remained at least 60.
(2) Validation was considered successful if at least 90% of measurements (54 of 60) fell within the proposed reference interval.
(3) If the initial validation pass rate was below 90%, the same procedure was repeated in a second validation round.
(4) The reference interval was considered successfully validated if the 90% criterion was met in the second round; otherwise, validation was deemed unsuccessful.

### 2.8 Outlier identification and handling procedure

Outliers were identified and removed using Tukey’s method as recommended by WS/T 408–2024 (15). The lower and upper limits were defined using the first quartile (Q1), third quartile (Q3), and interquartile range (IQR = Q3–Q1), according to the following formulas: lower limit = Q1–1.5 × IQR and upper limit = Q3+1.5 × IQR. Any value outside these limits was regarded as an outlier and excluded. The process was repeated iteratively until all remaining observations fell within the allowable range. If the sample size in any subgroup fell below 120 after outlier removal, additional participants were recruited to restore the minimum required number. After the final exclusion process, all subgroups still contained more than 120 individuals, meeting the guideline requirement. Outlier removal was performed primarily to reduce the influence of extreme values on percentile estimation and thereby improve the robustness and standardization of the statistical analysis.

### 2.9 Statistical analysis

Statistical analyses were performed using SPSS Statistics version 19.0 (IBM, Armonk, NY, United States). Normality was assessed with the Kolmogorov-Smirnov test. Normally distributed data are presented as mean ± standard deviation, whereas non-normally distributed data are expressed as median (M) and interquartile range (IQR). Group comparisons were performed using nonparametric methods, specifically the Mann-Whitney U test for two-group comparisons and the Kruskal-Wallis test followed by Dunn’s post hoc test for multiple-group comparisons. Correlations were analyzed using Spearman’s rank correlation coefficient. Reference intervals were established using the nonparametric 95th percentile. The order statistics approach was employed for percentile calculation, with each percentile taken as the [0.95 × (n + 1)] th order statistic. A two-sided *P* < 0.05 was considered statistically significant.

## 3 Results

### 3.1 Participant demographics and laboratory profiles

After application of the inclusion and exclusion criteria, 728 individuals were included in the final reference cohort. Demographic variables, including sex, age, and BMI, together with routine laboratory parameters (WBC, CRP, ALT, AST, urea, creatinine, GLU, TC, and TG), were collected for all participants. The complete dataset is provided in Supplementary Table 2.

### 3.2 Correlation analysis between cytokines and routine indicators

**FIGURE 2:**
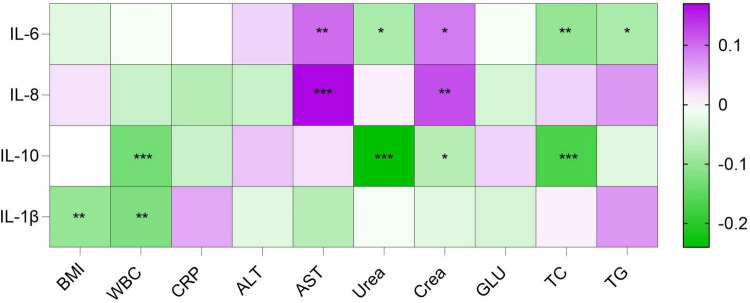
Correlation heatmap between cytokines and routine indicators Spearman correlation coefficients are shown. Green indicates negative correlation, purple indicates positive correlation, white indicates zero correlation. *p < 0.05, **p < 0.01, ***p < 0.001.

Spearman’s rank correlation analysis was performed to assess the relationships between the four cytokines (IL-6, IL-8, IL-10, and IL-1β) and routine laboratory indicators, including BMI, WBC, CRP, ALT, AST, urea, creatinine, GLU, TC, and TG. All observed correlations were weak, with correlation coefficients below 0.3. Detailed results are shown in Figure 2.

### 3.3 Distribution of plasma cytokines by sex and age

**FIGURE 3:**
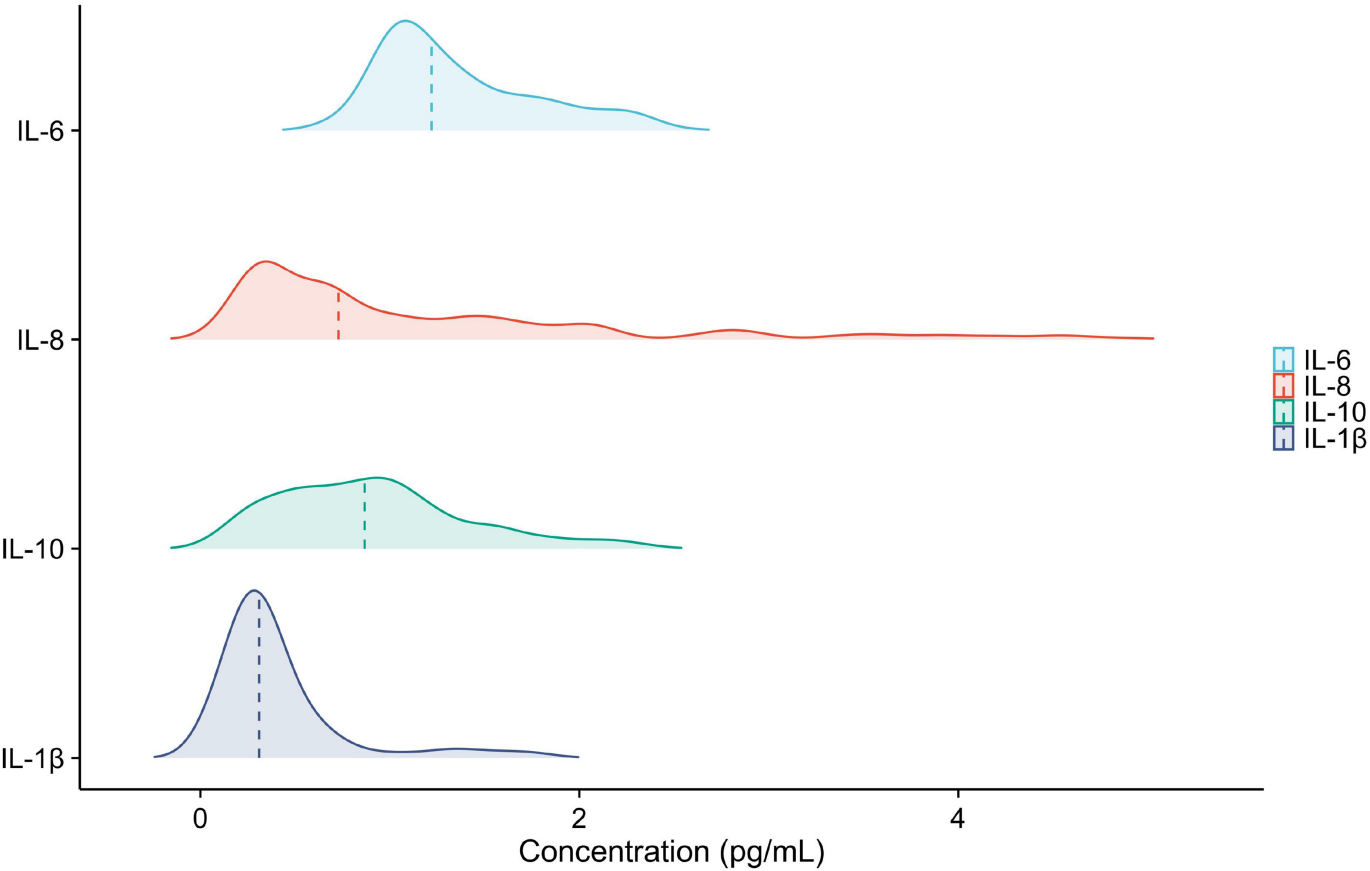
Distribution of plasma IL-6, IL-8, IL-10, and IL-1β levels. The *P*-values from the Kolmogorov-Smirnov test were all less than 0.05.

The Kolmogorov-Smirnov test showed that plasma IL-6, IL-8, IL-10, and IL-1β concentrations were not normally distributed in the study population (Figure 3).

**FIGURE 4:**
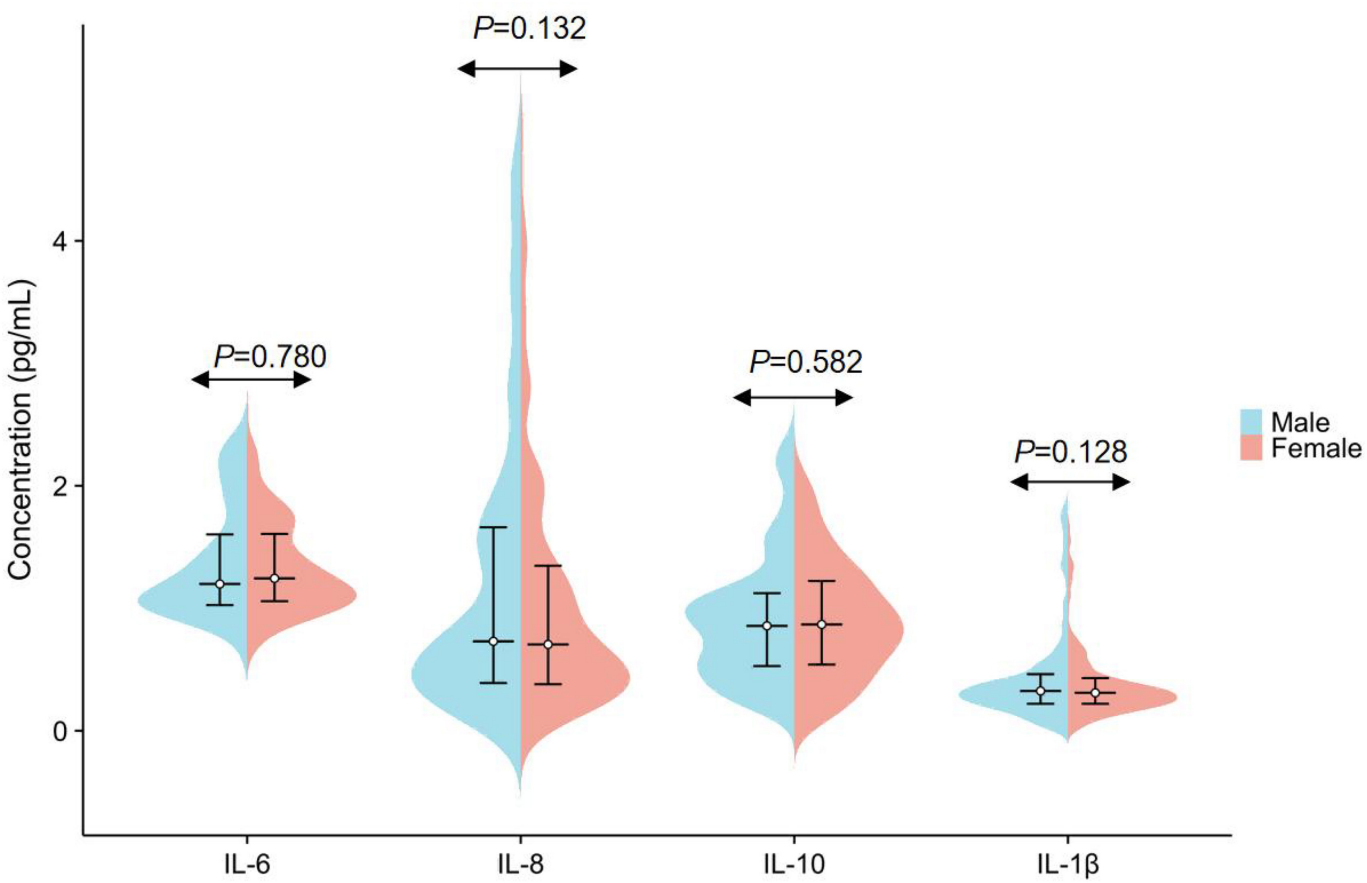
Sex-based differences in plasma IL-6, IL-8, IL-10, and IL-1β. The levels of IL-6, IL-8, IL-10, and IL-1β are presented as median and interquartile range.

**FIGURE 5:**
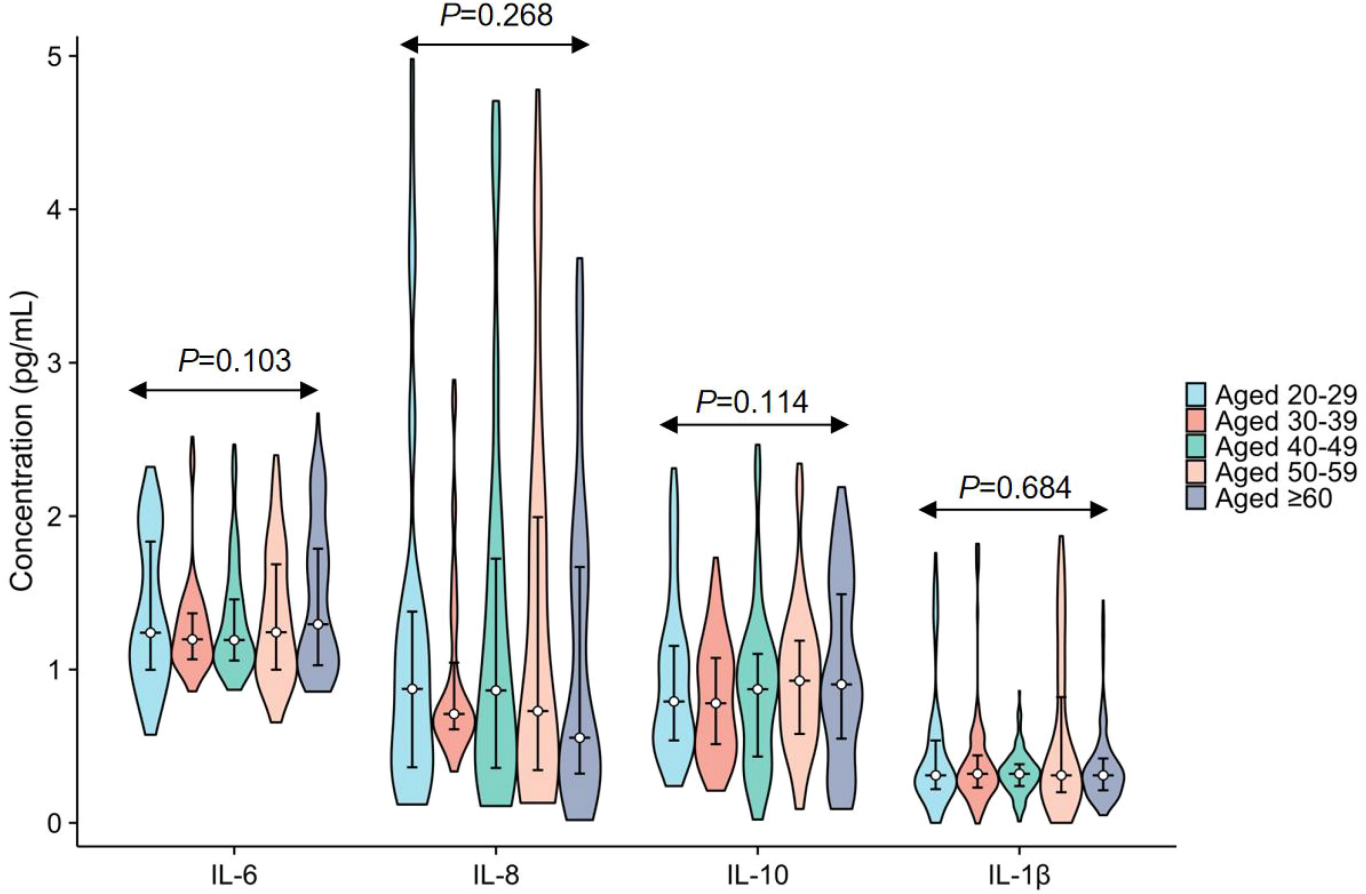
Age-related variations in plasma IL-6, IL-8, IL-10, and IL-1β. The levels of IL-6, IL-8, IL-10, and IL-1β are presented as median and interquartile range.

Initial comparisons between men and women showed no statistically significant sex-related differences for any of the four cytokines (all *P* > 0.05; Figure 4 and Table 1). Participants were then divided into five age groups, and no significant differences in IL-6, IL-8, IL-10, or IL-1β concentrations were observed across these groups (all *P* > 0.05; Figure 5 and Table 2).

**TABLE 1:** Sex-based differences in plasma IL-6, IL-8, IL-10, and IL-1β.

Analytes	Total (*n* = 728)	Gender groups		*P*-value
		Male (*n* = 364)	Female (*n* = 364)	
IL-6 (pg/mL)	1.22 (1.04–1.61)	1.20 (1.03–1.61)	1.25 (1.06–1.61)	0.780
IL-8 (pg/mL)	0.73 (0.39–1.57)	0.73 (0.39–1.66)	0.71 (0.38–1.35)	0.132
IL-10 (pg/mL)	0.87 (0.53–1.17)	0.86 (0.53–1.13)	0.87 (0.54–1.22)	0.582
IL-1β (pg/mL)	0.31 (0.22–0.44)	0.33 (0.22–0.47)	0.31 (0.22–0.43)	0.128

IL-6, Interleukin-6; IL-8, Interleukin-8; IL-10, Interleukin-10; IL-1β, Interleukin-1β. The indicators of IL-6, IL-8, IL-10, and IL-1β were represented as the median (M) and interquartile range (IQR).

**TABLE 2:** Age-related variations in plasma IL-6, IL-8, IL-10, and IL-1β.

Analytes	Age groups					*H*-value	*P*-value
	20–29 years (*n* = 126)	30–39 years (*n* = 147)	40–49 years (*n* = 140)	50–59 years (*n* = 133)	≥ 60 years (*n* = 182)		
IL-6 (pg/mL)	1.24 (1.00–1.85)	1.20 (1.07–1.37)	1.20 (1.06–1.47)	1.24 (1.00–1.70)	1.30 (1.03–1.80)	7.710	0.103
IL-8 (pg/mL)	0.87 (0.36–1.38)	0.71 (0.61–1.05)	0.86 (0.35–1.73)	0.73 (0.34–2.00)	0.56 (0.32–1.68)	5.191	0.268
IL-10 (pg/mL)	0.79 (0.54–1.16)	0.78 (0.51–1.08)	0.87 (0.43–1.11)	0.93 (0.57–1.21)	0.90 (0.55–1.49)	7.449	0.114
IL-1β (pg/mL)	0.31 (0.22–0.54)	0.32 (0.23–0.44)	0.32 (0.24–0.39)	0.31 (0.20–0.85)	0.31 (0.21–0.42)	2.281	0.684

IL-6, Interleukin-6; IL-8, Interleukin-8; IL-10, Interleukin-10; IL-1β, Interleukin-1β. The indicators of IL-6, IL-8, IL-10, and IL-1β were represented as the median (M) and interquartile range (IQR).

Because no significant age-related differences were identified, post hoc pairwise comparisons were not performed. Effect size analysis was also used to support decisions regarding stratification. Cohen’s d was calculated for sex-based comparisons, and the correlation coefficient (r) was used for age-related comparisons. All Cohen’s d values were below 0.2 and all r values were below 0.1, indicating consistently small effects. Therefore, stratification by sex or age was considered unnecessary. Detailed effect size estimates are listed in Table 3.

**TABLE 3:** Effect sizes of sex or age on plasma cytokine concentrations.

Parameter	Cytokines	Cohen’s *d* */*r*-value**	Evaluation results
Gender	IL-6	0.07	Small effect
	IL-8	0.17	Small effect
	IL-10	0.01	Small effect
	IL-1β	0.17	Small effect
Age	IL-6	0.09	Small effect
	IL-8	0.04	Small effect
	IL-10	0.08	Small effect
	IL-1β	0.03	Small effect

IL-6, Interleukin-6; IL-8, Interleukin-8; IL-10, Interleukin-10; IL-1β, Interleukin-1β. *d < 0.2 indicates a small effect; 0.20 ≤ *d* < 0.50 indicates a small to medium effect; 0.50 ≤ *d* < 0.80 indicates a medium effect; *d* ≥ 0.80 indicates a large effect. **r < 0.10 indicates a small effect; 0.10 ≤ *r* < 0.30 indicates a small to medium effect; 0.30 ≤ r < 0.50 indicates a medium effect; *r* ≥ 0.50 indicates a large effect.

### 3.4 Establishment of plasma cytokine reference intervals

Because no significant demographic stratification was required, all participants were analyzed as a single reference population for interval estimation. Following CLSI C28-A3 and WS/T 402–2024, upper reference limits were established using the nonparametric 95th percentile. The resulting reference intervals and 90% confidence intervals (CIs) for this regional Chinese population were IL-6 ≤ 2.23 pg/mL (90% CI: 2.18–2.27 pg/mL), IL-8 ≤ 3.76 pg/mL (90% CI: 3.51–3.98 pg/mL), IL-10 ≤ 1.89 pg/mL (90% CI: 1.81–2.02 pg/mL), and IL-1β ≤ 1.34 pg/mL (90% CI: 1.17–1.39 pg/mL). Detailed data are listed in Table 4.

**TABLE 4:** Distribution of plasma IL-6, IL-8, IL-10, and IL-1β in the entire cohort.

Analytes*	Distribution percentile (*n* = 728)			
	P_25_ (90% CI)	P_50_ (90% CI)	P_75_ (90% CI)	P_95_ (90% CI)
IL-6 (pg/mL)	1.04 (1.02–1.06)	1.22 (1.19–1.26)	1.61 (1.54–1.69)	2.23 (2.18–2.27)
IL-8 (pg/mL)	0.39 (0.36–0.41)	0.73 (0.69–0.78)	1.57 (1.48–1.66)	3.76 (3.51–3.98)
IL-10 (pg/mL)	0.53 (0.51–0.55)	0.87 (0.82–0.91)	1.17 (1.13–1.22)	1.89 (1.81–2.02)
IL-1β (pg/mL)	0.22 (0.20–0.23)	0.31 (0.30–0.33)	0.44 (0.42–0.47)	1.34 (1.17–1.39)

IL-6, Interleukin-6; IL-8, Interleukin-8; IL-10, Interleukin-10; IL-1β, Interleukin-1β; P_25_, 25th percentile; P_50_, 50th percentile; P_75_,75th percentile; P_95_, 95th percentile; CI, confidence interval. * When the plasma cytokine concentration falls below the lower limit of detection (LLoD), results should be reported as “< LLoD.”

### 3.5 Validation of established reference intervals

To verify the proposed reference intervals, 60 apparently healthy individuals with an equal sex distribution and an age range of 20–79 years were enrolled as an independent validation cohort. More than 90% of the measurements for IL-6, IL-8, IL-10, and IL-1β fell within the corresponding proposed intervals (Table 5), supporting the suitability of these intervals for clinical application.

**TABLE 5:** Validation of established reference intervals.

Indicators	Reference intervals (pg/mL)	Number of reference individuals (n)	Proportion of results in the reference interval (%)	Evaluation criteria (%)	Conclusion (Pass or fail)
IL-6	≤ 2.23	60	93.3	≥ 90	Pass
IL-8	≤ 3.76	60	90.0	≥ 90	Pass
IL-10	≤ 1.89	60	96.7	≥ 90	Pass
IL-1β	≤ 1.34	60	98.3	≥ 90	Pass

IL-6, Interleukin-6; IL-8, Interleukin-8; IL-10, Interleukin-10; IL-1β, Interleukin-1β.

## 4 Discussion

Cytokine measurement is commonly performed using enzyme-linked immunosorbent assay, chemiluminescence, and flow cytometry (20–22). Each method has distinct advantages, but flow cytometry offers several notable strengths for multiplex cytokine profiling, including low sample volume requirements, high throughput, good analytical performance, and the ability to quantify multiple cytokines simultaneously (23, 24). For these reasons, it has been increasingly adopted in clinical laboratories.

At present, many laboratories still use reference intervals supplied by manufacturers. However, these intervals often have important limitations, including relatively small reference cohorts, typically fewer than 120 individuals, and insufficient stratified analysis by sex and age. As a result, their precision and clinical applicability may be limited. In the present study, flow cytometry was used, according to the manufacturer’s protocol, to quantify plasma IL-6, IL-8, IL-10, and IL-1β in healthy adults from Lianyungang, Jiangsu, eastern China. Based on this regional single-center dataset, we sought to improve the clinical interpretability of these biomarkers by establishing population-specific reference intervals. Using strict selection criteria, we enrolled 728 qualified reference individuals. Correlation analysis showed only weak associations between the four cytokines and routine laboratory parameters. In addition, the Kolmogorov-Smirnov test confirmed that plasma IL-6, IL-8, IL-10, and IL-1β concentrations were non-normally distributed in this healthy population. A major objective of the study was to evaluate the potential influence of demographic factors. After stratification by sex and into five age groups, nonparametric analyses showed no significant differences in the concentrations of any of the four cytokines. Effect size analysis further demonstrated that the effects of sex and age were consistently small. These findings suggest that, within this adult population, these cytokines show limited variation according to sex or age, supporting the use of unified reference intervals.

Our observations regarding the lack of sex-related differences are generally consistent with previous studies, although some discrepancies have been reported. Wu et al. (25), using chemiluminescence, found no significant sex-based differences in serum IL-1β, IL-2γ, IL-6, IL-8, or IL-10 among 180 healthy adults. Likewise, Lan et al. (26), using flow cytometry in 100 individuals, reported no sex-related differences in a broader cytokine panel including IFN-γ, TNF-α, IL-2, IL-4, IL-6, IL-10, and IL-17A. Huang et al. (27) also found no sex-related difference for IL-1β, IL-6, or IL-10 using chemiluminescence; however, they reported higher IL-8 and TNF-α levels in men. These inconsistencies suggest that the pattern may vary depending on the analyte and analytical platform. Notably, most previous studies were limited by relatively small sample sizes and did not formally evaluate age-related trends when establishing reference intervals. In contrast, our study systematically assessed both sex and age in a substantially larger, region-specific cohort, thereby strengthening the robustness and representativeness of the proposed intervals.

For many years, clinical laboratories have depended on previously established reference intervals, primarily because they are easy to obtain and convenient for routine use (28, 29). However, the appropriateness of this practice has come under increasing scrutiny. One major issue is that generalized reference intervals may fail to accurately reflect the characteristics of local populations. Biological and social determinants—including ethnicity, sex, and age—are known to affect the physiological ranges of numerous biomarkers (28, 29). Therefore, the use of unvalidated external reference intervals can result in diagnostic misclassification. In response, the harmonization of reference interval establishment has become a prominent and widely discussed topic in laboratory medicine in recent years (30–32). This drive toward higher precision is also evident in national efforts. For instance, the National Health Commission of the People’s Republic of China has released evidence-based reference intervals for common laboratory analytes derived from multicenter population studies, thereby offering a crucial framework for regional adjustments (33, 34). Following this rationale and adhering to CLSI C28-A3 and WS/T 402–2024 guidelines, the present study established and validated reference intervals for plasma IL-6, IL-8, IL-10, and IL-1β in healthy adults from Lianyungang, Jiangsu, eastern China.

The intervals established in the present study were validated in an independent cohort of 60 healthy individuals, and more than 90% of values fell within the proposed limits, confirming their acceptability. Compared with previous reports, however, the intervals observed here differ to varying degrees. Zhang et al. (35), in their evaluation of a flow cytometry platform, reported manufacturer-supplied limits such as IL-6 ≤ 5.4 pg/mL, which are higher than the upper limit identified in our study. Lan et al. (26) also reported different ranges for IL-6 and IL-10 in their single-center study. An even more marked difference was noted in the study by Adedeji et al. (36), who used high-performance liquid chromatography in a Nigerian population and reported reference intervals in ng/L. The discrepancies between the aforementioned reports and the present study may be attributable to the following factors: (1) Differences in study populations. The present study involved healthy Han Chinese adults from the eastern region of Jiangsu Province, whose ethnic background, dietary patterns (e.g., high-salt intake and high seafood consumption), and living environment exhibit notable regional characteristics. (2) Systematic differences in results obtained from different detection platforms (e.g., flow cytometry, chemiluminescence, enzyme-linked immunosorbent assay). Therefore, integrating the findings from previous reports and the current study, reference intervals should not be directly transferred without distinction across different regions or detection systems. Instead, external reference intervals should be validated, or appropriate intervals should be established locally, based on the laboratory’s own population characteristics, detection methods, and workflows, to ensure that test results are clinically meaningful.

Several limitations should be acknowledged. First, this was a single-center study, and participants were recruited primarily from the eastern coastal region of China, which may limit the representativeness of the results. Accordingly, laboratories intending to adopt these intervals should perform local validation in larger populations. Second, the study included only adults aged 20–79 years and did not include infants, children, or adolescents; therefore, the proposed intervals should not be extrapolated to younger age groups without formal verification. Third, although each subgroup met the minimum guideline requirement of 120 individuals, the overall sample size remains moderate. Because flow cytometric cytokine measurement is relatively costly, recruitment of a larger cohort was not feasible in the present study. Future multicenter studies involving larger populations and broader age coverage are warranted to establish more representative and scientifically robust cytokine reference intervals and to further clarify their value in clinical decision-making.

## 5 Conclusion

In summary, this study established preliminary reference intervals for plasma IL-6, IL-8, IL-10, and IL-1β in healthy adults from Lianyungang, Jiangsu, eastern China, using flow cytometry. These intervals may serve as a useful tool for assessing individual immune status and may support the diagnosis and clinical management of related diseases.

## Data Availability

The raw data supporting the conclusions of this article will be made available by the authors, without undue reservation.
