# Supplementary material for: Establishment of reference intervals for plasma IL-6, IL-8, IL-10, and IL-1β in healthy adults from Lianyungang, Jiangsu, China: a single-center flow cytometry analysis
Source: Front Med (Lausanne). 2026 May 13;13:1789886. doi: 10.3389/fmed.2026.1789886 (PMC13212108; doi:10.3389/fmed.2026.1789886)
Supplement: Supplementary file 1 [file Table_1.doc]

**Supplementary Table 1.** Performance Characteristics and Verification Results of Cytokine Assays

| Performance Parameters | Manufacturer’s specifications | Laboratory Verification Results | Conclusion  (Pass or fail) |
| --- | --- | --- | --- |
| LoB (pg/mL) | 0.20 pg/mL | 0.20 pg/mL | Pass |
| LoD (pg/mL) | 0.50 pg/mL | 0.50 pg/mL | Pass |
| LLoQ (pg/mL) | 1.00 pg/mL | 1.00 pg/mL | Pass* |
| Intra-assay precision | 8.00% | IL-6：2.69% (Level 1) and 3.45% (Level 2)  IL-8：3.02% (Level 1) and 6.55% (Level 2)  IL-10：7.44% (Level 1) and 3.24% (Level 2)  IL-1β：6.81% (Level 1) and 5.34% (Level 2) | Pass** |
| Inter-assay precision | 12.00% | IL-6：4.17% (Level 1) and 4.64% (Level 2)  IL-8：5.27% (Level 1) and 8.10% (Level 2)  IL-10：8.82% (Level 1) and 3.24% (Level 2)  IL-1β：7.52% (Level 1) and 6.75% (Level 2) | Pass*** |
| Trueness | 15% | IL-6：4.68% (Level 1) and 2.28% (Level 2)  IL-8：4.86% (Level 1) and 5.72% (Level 2)  IL-10：2.31% (Level 1) and 1.71% (Level 2)  IL-1β：5.41% (Level 1) and 6.25% (Level 2) | Pass |
| Linearity | 1.00-10,000.00 pg/mL  (R^2^≥0.995) | IL-6：1.04-7,595.55 pg/mL (R^2^=0.999)  IL-8：1.06-7,844.54 pg/mL (R^2^=0.999)  IL-10：1.07-7,546.51 pg/mL (R^2^=0.999)  IL-1β：1.04-7,362.51 pg/mL (R^2^=0.998) | Pass**** |
| Reportable range | 1.00-20,000.00 pg/mL | IL-6：1.04-15,191.10 pg/mL  IL-8：1.06-15,689.08 pg/mL  IL-10：1.07-15,093.02 pg/mL  IL-1β：1.04-14,724.82 pg/mL | Pass***** |

IL-6: Interleukin-6; IL-8: Interleukin-8; IL-10: Interleukin-10; IL-1β: Interleukin-1β; LoB: limit of blank; LoD: limit of detection; LLOQ: lower limit of quantification.

* Indicates that when cytokine concentrations fall below the lower limit of quantification (LLOQ, ≤1.00 pg/mL), most healthy individuals exhibit levels at or below the precise quantitation threshold of the assay. Clinically, results reported as <LLOQ (<1.00 pg/mL) are regarded as falling within the expected range for healthy subjects, whereas measurable concentrations (≥1.00 pg/mL) may warrant further assessment for possible immune activation. Notably, the clinical relevance of cytokines derives primarily from elevated levels, whereas the LLOQ itself offers limited diagnostic or therapeutic value.

*** Indicates that validation was conducted at two distinct concentration levels: Level 1 (low concentration) and Level 2 (high concentration).

**** Refers to cytokine concentrations verified in our laboratory using fresh samples exhibiting abnormally high values. Since samples matching the manufacturer's declared high-value concentration (10,000.00 pg/mL) were unavailable, the verified concentrations are reported as shown in the table.

***** Denotes that the verified upper limits satisfy clinical application requirements. When concentrations exceed these limits, results are reported as above the upper limit of detection (IL-6 > 15,191.10 pg/mL; IL-8 > 15,689.08 pg/mL; IL-10 > 15,093.02 pg/mL; IL-1β > 14,724.82 pg/mL).

**Supplementary Table 2.** Participant demographics and laboratory profiles

| Indicators | All  (n=728) | Male  (n=364) | Female  (n=364) | Reference interval |
| --- | --- | --- | --- | --- |
| Age (years) | 48 (33, 60) | 48 (32, 58) | 47 (35, 62) | - |
| BMI (kg/m^2^) | 23.2±2.3 | 23.5±2.0 | 22.8±2.6 | 18.5-27.9 |
| WBC (×10^9^/L) | 5.6±1.4 | 5.6±1.6 | 6.2±1.3 | 3.5-9.5 |
| CRP (mg/L) | 2.36 (1.06, 4.49) | 2.99 (1.52, 4.85) | 2.29 (1.23, 4.23) | 0.00-10.00 |
| ALT (U/L) | 23.9±7.7 | 25.3±7.1 | 22.4±8.0 | 9.0-50.0 (male)  7.0-40.0 (female) |
| AST (U/L) | 23.8±6.8 | 26.7±6.6 | 20.9±5.7 | 15.0-40.0 (male)  13.0-35.0 (female) |
| Urea (mmol/L) | 5.6±1.4 | 5.4±1.1 | 5.9±1.6 | 3.1-8.0 (male, aged 20-59 years)  3.6-9.5 (male, aged 60-79 years)  2.6-7.5 (female, aged 20-59 years)  3.1-8.8 (female, aged 60-79 years) |
| Creatinine (µmol/L) | 67±14 | 77±11 | 58±10 | 57-97 (male, aged 20-59 years)  57-111 (male, aged 60-79 years)  41-73 (female, aged 20-59 years)  41-81 (female, aged 60-79 years) |
| GLU (mmol/L) | 4.80±0.52 | 4.81±0.52 | 4.80±0.52 | 3.89-6.11 |
| TC (mmol/L) | 4.34±0.47 | 4.54±0.37 | 4.15±0.48 | <5.17 |
| TG (mmol/L) | 0.93±0.25 | 0.96±0.24 | 0.90±0.25 | <1.70 |

BMI: body mass index; WBC: white blood cell count; CRP: C-reactive protein; ALT: alanine aminotransferase; AST: aspartate aminotransferase; GLU: glucose;TC: total cholesterol; TG: triglycerides. The indicators of age and CRP were represented as the median (M) and interquartile range (IQR); the indicators of BMI, WBC, ALT, AST, Urea, Crea, GLU, TC and TG were represented by the mean ± standard deviation.
